# Measurement properties of physical activity in adults with bronchiectasis: A systematic review protocol

**DOI:** 10.12688/f1000research.138593.1

**Published:** 2023-07-10

**Authors:** Anup Bhat, Annemarie L Lee, G Arun Maiya, K Vaishali

**Affiliations:** 1 Department of Physiotherapy, Manipal College of Health Professions, Manipal Academy of Higher Education, Manipal, Karnataka, 576104, India; 2 Department of Physiotherapy, School of Primary and Allied Health Care, Monash University, Clayton, Frankston, Victoria, 3199, Australia

**Keywords:** Bronchiectasis, exercise, movement, outcome assessment, physical exertion

## Abstract

**Abstract: **People with bronchiectasis reduce their physical activity (PA) due to muscle weakness, dyspnea, fatigue, reduced exercise capacity and frequent cough with expectoration. Patient-reported and objective physical assessment methods have been used to evaluate PA in people with bronchiectasis. In the literature, significant differences in the PA measured using patient-reported outcome measures when compared with the objective methods. Given the availability of many PA assessment tools, it is tedious for the clinician or researcher to choose an outcome measure for clinical practice or research. The evidence on validity and reliability in bronchiectasis are unclear.

**Objectives:** To identify the PA assessment tools, describe and evaluate the literature on psychometric properties of instruments measuring and analyzing PA.

**Methods: **The search will be conducted in PubMed/Medline, Cochrane Central Register of Controlled Studies, Scopus and EMBASE databases. The keywords, index terms and synonyms of the following words will be used: bronchiectasis, physical activity, and outcome measures. Published studies of adult with clinical and/ or radiologically diagnosed bronchiectasis, aged >18 years, any gender and studies that assessed PA and/or if there are reports on measurement properties of PA will be included in the review. Studies using qualitative research methods, narrative reviews, letters to editors and editorials will be excluded. The quality of the study will be assessed and data will be extracted. Any disagreement will be resolved in the presence of an author not involved in the screening or selecting studies.

**Discussion:** By assessing the quality of studies on measurement properties, this review will help researchers choose the outcome measure to evaluate the effects of interventions on PA. This review will identify the suite of outcome measures of PA for people with bronchiectasis that can be used for research and clinical purpose.

## Introduction

Bronchiectasis is a chronic airway disease which is marked by chronic cough with expectoration, and recurrent exacerbations. ^1^^,^^2^ Clinical presentations may differ based on the underlying cause of bronchiectasis. ^2^ Muscular weakness, loss of muscular endurance, dyspnea, fatigue and poor quality of life are major clinical features experienced by people with bronchiectasis. ^3^ These factors collectively contribute to reduced exercise capacity and limited participation in physical activity (PA). ^3^^–^^5^ Physical activity is any movement caused by muscular contraction resulting in energy consumption. ^6^ To reduce symptoms of dyspnea, those with bronchiectasis often avoid activities of daily living, which result in further deconditioning. ^7^^,^^8^ In addition, the frequency of coughing and sputum expectoration in people with bronchiectasis may increase with PA. ^9^ As a consequence, the social stigma associated with chronic cough and sputum expectoration in public is associated with frustration and embarrassment to people with bronchiectasis. ^10^ To avoid social embarrassment associated with coughing bouts, people with bronchiectasis often reduce their PA. ^9^^–^^11^ José *et al*., assessed PA in the stable adult bronchiectasis population using pedometers and showed significant reduction in step counts compared to healthy age-matched peers. ^6^ Similarly, Cakmak *et al.,* demonstrated that both objectively measured PA using multisensorial PA monitor and through self-reported questionnaire in the stable adult bronchiectasis population was lower than age-matched healthy individuals. ^12^ Furthermore, the study highlighted the significant difference between objective and subjective methods of PA assessment. Duration of PA measured through objective method was higher than those obtained from subjective method. ^12^ O’Neil *et al.*, compared pedometer and a questionnaire with the criterion assessment tool accelerometer in people with stable bronchiectasis. The physical activity in terms of step counts between pedometer and accelerometer were comparable; however, the questionnaire over-reported the moderate-vigorous physical activity (MVPA) compared with the accelerometer. ^13^ Considering the lower level of PA in people with bronchiectasis compared to aged-matched peers, it is important for the PA assessment tool to be sensitive enough to appreciate small changes. ^12^ Physical activity has an impact on the prognosis of bronchiectasis ^14^; those with lower PA levels had higher risk of hospitalization. ^14^ Increased rate of exacerbation was associated with reduced PA levels and higher sedentary behaviour in adults with bronchiectasis. ^15^

In order to assess PA, a wide range of tools are available. These include patient (self) reported outcome measures (PROMs) such as global physical activity questionnaire (GPAQ), recent physical activity questionnaires, international physical activity questionnaire (IPAQ), and self-report activity diaries/logs. ^16^ Alternatively, there are objective methods such as direct observation and device-based measurements (accelerometers, pedometers, heart rate monitors and armbands). ^16^ Recently, mobile phone-based applications are also used to measure PA. ^17^

Physical activity is an important outcome in pulmonary rehabilitation (PR) as PR is expected to improve movement efficiency, cardiovascular function and skeletal muscle oxidative function, thereby enhancing PA. ^18^ Only a handful of studies have evaluated PA following PR for people with bronchiectasis. ^19^^–^^21^ Given the availability of many PA assessment tools, it is a tedious task for the clinician or researcher to choose an outcome measure for clinical practice or research. The evidence on validity and reliability in bronchiectasis are unclear. Therefore, it is important to identify tools that are used to measure PA in people with bronchiectasis and discuss their measurement properties. Furthermore, it is important to identify the feasibility, cost and their limitations. The measurement properties of PA measurement tools examined in this systematic review may inform clinicians and researchers regarding the availability of various PA assessment tools and their validity and reliability, enabling appropriate choices for practice.

To address the current knowledge gap, this systematic review aims to identify and evaluate the measurement properties of PA assessment tools which have been applied in people with bronchiectasis. The specific objectives of the systematic review are to 1) identify the approaches used to measure PA in bronchiectasis; 2) describe and evaluate the literature available on psychometric properties, including validity, reliability, responsiveness and interpretability of instruments measuring and analysing PA; and 3) provide recommendations on most suitable and effective ways of measuring PA in bronchiectasis.

## Methods

The review is registered on PROSPERO, an international prospective register of systematic reviews (CRD42023423087). This systematic review protocol will follow the Preferred Items for Systematic Reviews and Meta-Analyses Protocols (PRISMA-P) reporting guidelines. ^22^ Preferred Reporting Items for Systematic Reviews and Meta-Analyses (PRISMA) guidelines will be used for subsequent systematic review. ^23^

### Study eligibility criteria

Inclusion criteria: Published studies of adult with clinical and/ or radiologically diagnosed bronchiectasis, ^24^ aged ≥18 years, any gender, geographical location and studies that assessed PA and/or if there are reports on measurement properties of PA listed in COnsensus-based Standards for the selection of health Measurement Instruments (COSMIN) taxonomy of measurement properties. ^25^ The review will be limited only to the English language. Studies using mixed respiratory pathology will be included if at least 80% of the included people had primary diagnosis of bronchiectasis or data of sub-group are reported separately for the bronchiectasis population. Studies that include mixed populations (such as adult and paediatric) will also be included only if the data on adult bronchiectasis are provided separately. Studies investigating both acute exacerbations and those in stable conditions will be included. Any reviews of potential relevance to the topic will be screened to identify potentially eligible studies included within that review.

Exclusion criteria: Studies using qualitative research methods, narrative review, letters to editors and editorials will be excluded. Studies that report other constructs related to PA such as physical function, mobility, functional status, and activities score from health-related quality of life measures, and activities of daily living will be excluded. Studies that report PA in people with cystic fibrosis will be excluded.

The definitions of each measurement properties from COSMIN taxonomy of measurement properties ^25^ are listed in Table 1.

**Table 1.** Definitions of domain and measurement properties as per COSMIN taxonomy of measurement properties [adapted from Mokkink *et al*. ^25^] (copyright obtained Elsevier, license number 5570070383945).

Domain	Measurement property	Definition
Validity		The degree to which an HR-PRO or performance-based instrument measures the construct(s) it purports to measure.
	Content validity	The degree to which the content of an HR-PRO instrument is an adequate reflection of the construct to be measured. This includes face validity.
	Construct validity	The degree to which the scores of an HR-PRO/performance-based instrument are consistent with hypotheses based on the assumption that the HR-PRO instrument validly measures the construct to be measured. This includes structural validity, cross-cultural validity and hypothesis testing.
	Criterion validity	The degree to which the scores of an HR-PRO/performance-based instrument are an adequate reflection of a “gold standard”.
Reliability	Reliability	The degree to which the measurement is free from measurement error. The extent to which scores for patients who have not changed are the same for repeated measurement under several conditions such as test-retest, inter-rater and intra-rater measurements.
	Internal consistency	The degree of the interrelatedness among the items.
	Measurement error	The systematic and random error of a patient’s score that is not attributed to true changes in the construct to be measured.
Responsiveness	Responsiveness	The ability of an HR-PRO/performance-based instrument to detect change over time in the construct to be measured.
Interpretability		The degree to which one can assign qualitative meaning that is, clinical or commonly understood connotations to an instrument’s quantitative scores or change in scores.

Abbreviations: HR-PRO: Health related patient reported outcomes.

### Search strategy and terms

The search will be conducted in PubMed/Medline, Cochrane Central Register of Controlled Studies (CENTRAL), Scopus and EMBASE databases. The keywords that will be used for the search are non-cystic fibrosis bronchiectasis, bronchiectasis, physical activity, questionnaire, self-reported physical activity, pedometer, activity tracker. Search strategy for PubMed database is presented in Table 2. Search filters for PubMed and EMBASE will be developed based on the methods suggested by Terwee *et al*. ^26^ The references from all the included articles will be screened to identify additional articles.

**Table 2.** Search strategy for PubMed/Medline.

Search concepts	Keywords
#1	((((((((((((((((((((((((((((((((“Yellow nail syndrome*”) OR (“yellow nail syndrome*”[Text Word])) OR (“yellow nail syndrome*”[Title/Abstract])) OR (syndrome, yellow nail [MeSH Terms])) OR (“primary ciliary dyskinesi*”)) OR (“primary ciliary dyskinesi*”[Text Word])) OR (“primary ciliary dyskinesi*”[Title/Abstract])) OR (dyskinesia, primary ciliary [MeSH Terms])) OR (“ciliary motility disorder*”)) OR (“ciliary motility disorder*”[Text Word])) OR (“ciliary motility disorder*”[Title/Abstract])) OR (“ciliary motility disorders”[MeSH Terms])) OR (bronchiect*)) OR (bronchiect*[Text Word])) OR (bronchiect*[Title/Abstract])) OR (bronchoectasia)) OR (bronchoectasia [Text Word])) OR (bronchoectasia [Title/Abstract])) OR (bronchiectasia)) OR (bronchiectasia [Text Word])) OR (bronchiectasia [Title/Abstract])) OR (non-cystic fibrosis*)) OR (non-cystic fibrosis*[Text Word])) OR (non-cystic fibrosis*[Title/Abstract])) OR (non-CF*)) OR (non-CF*[Text Word])) OR (non-CF*[Title/Abstract])) OR (NCFB)) OR (NCFB [Text Word])) OR (NCFB [Title/Abstract]) OR (“Kartagener Syndrome”[Mesh])) OR (“Kartagener syndrome”[Title/Abstract])) OR (“Kartagener syndrome”[Text Word]))
#2	(((((((((((((((((((((((((((((((((((((((((((((((((((((((((((((((((((((((((((((((((((((((((((((exercise [MeSH Terms]) OR (“exercise”[Title/Abstract])) OR (“exercise”[Text Word])) OR (exercise)) OR (“physical activit*”[Title/Abstract])) OR (“physical activit*”[Text Word])) OR (“physical activit*”)) OR (“motor activity”[MeSH Terms])) OR (“motor activit*”[Title/Abstract])) OR (“motor activit*”[Text Word])) OR (“motor activit*”)) OR (“leisure activities”[MeSH Terms])) OR (“leisure activit*”[Title/Abstract])) OR (“leisure activit*”[Text Word])) OR (“leisure activit*”)) OR (“leisure-related physical activit*”[Title/Abstract])) OR (“leisure-related physical activit*”[Text Word])) OR (“leisure-related physical activit*”)) OR (“physical functional performance”[MeSH Terms])) OR (“physical functional performance”[Title/Abstract])) OR (“physical functional performance”[Text Word])) OR (“physical functional performance”)) OR (“physical fitness”[MeSH Terms])) OR (“physical fitness”[Title/Abstract])) OR (“physical fitness”[Text Word])) OR (“physical fitness”)) OR (“sedentary behavior”[MeSH Terms])) OR (“sedentary Behavi*”[Title/Abstract])) OR (“sedentary Behavi*”[Text Word])) OR (“sedentary Behavi*”)) OR (“physical inactivity”[Title/Abstract])) OR (“physical inactivity”[Text Word])) OR (“physical inactivity”)) OR (“occupational physical activit*”[Title/Abstract])) OR (“occupational physical activit*”)) OR (“Occupational physical activit*”[Text Word])) OR (“health behavior”[MeSH Terms])) OR (“healthy behavi*”[Title/Abstract])) OR (“healthy behavi*”[Text Word])) OR (“healthy behavi*”)) OR (“leisure time physical activit*”[Title/Abstract])) OR (“leisure time physical activit*”)) OR (“leisure time physical activit*”[Text Word])) OR (“household physical activit*”[Title/Abstract])) OR (“household physical activit*”[Text Word])) OR (“household physical activit*”)) OR (“walking”[MeSH Terms])) OR (“walk*”[Title/Abstract])) OR (“walk*”[Text Word])) OR (“walk*”)) OR (running [MeSH Terms])) OR (“running”[Title/Abstract])) OR (“running”[Text Word])) OR (“running”)) OR (“video games”[MeSH Terms])) OR (“games, recreational”[MeSH Terms])) OR (“recreational game*”[Title/Abstract])) OR (“recreational game*”[Text Word])) OR (“recreational game*”[Text Word])) OR (“recreational game*”)) OR (“sports”[MeSH Terms])) OR (sports [Title/Abstract])) OR (sports [Title/Abstract])) OR (sports [Text Word])) OR (sports)) OR (“game*”[Title/Abstract])) OR (“game*”[Text Word])) OR (game*)) OR (gaming [Title/Abstract])) OR (gaming [Text Word])) OR (gaming)) OR (“bicycling”[MeSH Terms])) OR (“bicycl*”[Title/Abstract])) OR (“bicycl*”[Text Word])) OR (“bicycl*”)) OR (“physically active”[Title/Abstract])) OR (“Physically active”[Text Word])) OR (“physically active”)) OR (“exercise therapy”[MeSH Terms])) OR (“exercise therapy”[Title/Abstract])) OR (“exercise therapy”[Text Word])) OR (“exercise therapy”[Text Word])) OR (“exercise therapy”)) OR (“Healthy Lifestyle”[MeSH Terms])) OR (“Healthy Lifestyle”[Title/Abstract])) OR (“Healthy Lifestyle”[Text Word])) OR (“Healthy Lifestyle”)) OR (sitting [Title/Abstract])) OR (sitting [Text Word])) OR (sitting)) OR (“Health promotion”[MeSH Terms])) OR (“health promotion”[Title/Abstract])) OR (“health promotion”[Text Word])) OR (“health promotion”)
#3	(((((((((((((((((((((((((((((((((((((((((((((((((((((((((((((((((((((((((((((((MVPA) OR (MVPA [Text Word])) OR (MVPA [Title/Abstract])) OR (“Acceleromet*”)) OR (“Acceleromet*”[Text Word])) OR (“Acceleromet*”[Title/Abstract])) OR (Accelerometry [MeSH Terms])) OR (“fitness trackers”[MeSH Terms])) OR (“fitness tracker*”[Title/Abstract])) OR (“fitness tracker*”[Text Word])) OR (“fitness tracker*”)) OR (“Surveys and Questionnaires”[MeSH Terms])) OR (“questionnaire*”[Title/Abstract])) OR (“questionnaire*”[Text Word])) OR (“questionnaire*”)) OR (“actigraphy”[MeSH Terms])) OR (“actigraph*”[Title/Abstract])) OR (“actigraph*”[Text Word])) OR (“actigraph*”)) OR (“pedometer*”[Title/Abstract])) OR (“pedometer*”[Text Word])) OR (“pedometer*”)) OR (“activity monitor*”[Title/Abstract])) OR (“activity monitor*”[Text Word])) OR (“activity monitor*”)) OR (“motion detect*”[Title/Abstract])) OR (“motion detect*”[Text Word])) OR (“motion detect*”)) OR (“step count*”[Title/Abstract])) OR (“step count*”[Text Word])) OR (“step count*”)) OR (“movement detect*”[Text Word])) OR (“movement detect*”)) OR (“movement detect*”[Title/Abstract])) OR (“motion sens*”[Title/Abstract])) OR (“motion sens*”[Text Word])) OR (“motion sens*”)) OR (diary [Title/Abstract])) OR (diary [Text Word])) OR (diary)) OR (“diaries as topic”[MeSH Terms])) OR (“medical records”[MeSH Terms])) OR (“health diary”[Title/Abstract])) OR (“health diary”[Text Word])) OR (“health diary”)) OR (logs [Title/Abstract])) OR (logs [Text Word])) OR (logs)) OR (“self-report”[MeSH Terms])) OR (“self-report*”[Title/Abstract])) OR (“self-report*”[Text Word])) OR (“self-report*”)) OR (“observation”[MeSH Terms])) OR (“direct observation”[Title/Abstract])) OR (“direct observation”[Text Word])) OR (“direct observation”)) OR (“heart rate monitor*”[Title/Abstract])) OR (“heart rate monitor*”[Text Word])) OR (“heart rate monitor*”)) OR (“Patient Reported Outcome Measures”[MeSH Terms])) OR (“Patient Reported Outcome Measures”[Title/Abstract])) OR (“Patient Reported Outcome Measures”[Text Word])) OR (“Patient Reported Outcome Measures”)) OR (“performance based measures”[Title/Abstract])) OR (“performance based measures”[Text Word])) OR (“performance based measures”)) OR (“objective measure*”[Title/Abstract])) OR (“objective measure*”[Text Word])) OR (“objective measure*”)) OR (“subjective measure*”[Title/Abstract])) OR (“subjective measure*”[Text Word])) OR (“subjective measure*”[Text Word])) OR (“subjective measure*”)) OR (“Outcome Assessment, Health Care”[MeSH Terms])) OR (“outcome measure*”[Title/Abstract])) OR (“outcome measure*”[Text Word])) OR (“outcome measure*”)) OR (“measure*”[Title/Abstract])) OR (“measure*”[Text Word])) OR (“measure*”)
#4	**#1 AND #2 AND #3**

### Study selection, data extraction and quality assessment

*Selection procedure:*

The articles obtained from these databases will be collated in Rayyan and duplicates will be removed. Two authors (AB, VK) will independently screen the title and abstract of all the articles using Rayyan. ^27^ Any disagreements about inclusion will be resolved through discussions with (AL) as arbitrator. Interrater agreement (IRA) for titles and abstracts will be calculated. Full text articles will be independently reviewed by two authors (AB, VK) to finalize the full text articles which meet the inclusion criteria. Any disagreement will be resolved in the presence of (AL) as arbitrator.

Data extraction: A data extraction sheet will be prepared by one author (AB) and will be modified after the discussion with the entire team. Data extraction sheet will be created on Microsoft Excel spreadsheet by one author (AB). The data extraction sheet will contain the following

a. Demographic details of the participants such as age, gender, body mass index, severity of the bronchiectasis
b. Characteristics of the study such as name of the first author, country where the study was conducted, year of publication, study design, and sample size.
c. Characteristics of the outcome measure such as name of the measurement tool, abbreviation of the tool, self-reported or clinician administered, original reference for the tool, original language, available translations, recall period, score range, score interpretation, type of PA assessed, setting where it was administered such as hospital or home setting, cost and time taken to administer, equipment needed, training requirement, ease of score calculation, copyright, patient’s physical and mental ability level required to use and ease of standardisation.
d. Measurement properties of the scale: validity (includes content, construct, and criterion validity), reliability (includes reliability measures, measurement error and internal consistency), responsiveness and interpretability. If the psychometric properties have not been assessed in the included study, it will be entered as not applicable.

The form will be pretested on five studies by study authors prior to finalisation. One of the authors (AB) then will extract data from included studies, and a second author will confirm that the data were extracted correctly. Any discrepancies will be discussed with AL., and the final decision will be made through consensus.

Methodological quality assessment: Two independent reviewers (AB and VK) will rate the methodological quality of the included studies. Based on the design of the study, we will use CASP cohort checklist for cohort design ^28^ and Pedro scale for RCTs. ^29^ For studies including psychometric properties, the standardized COnsensus-based Standards for the selection of health Measurement INstruments (COSMIN) Risk of Bias checklist will be used. ^30^ The COSMIN methodology was specially developed and validated for the reviews of patient-reported outcome measures. A newer COSMIN Risk of Bias checklist has been developed for other types of outcome measurement instruments such as performance-based outcome measures, laboratory values and clinician-reported outcome measures. For these reasons, an adapted COSMIN Risk of Bias checklist on reliability and measurement error will be used for performance-based outcome measures. ^31^ Any discrepancies will be discussed with AL., and the final decision will be made through consensus. Methodological quality of each study will be tabulated in the summary of findings table.

Evaluation of outcome measure: The following measurement properties will be evaluated: validity (including content, criterion, construct validity), reliability (including internal consistency, test-retest, inter-rater, intra-rater reliability and measurement error), responsiveness and interpretability using COSMIN Risk of Bias checklists. ^30^^,^^31^

Data synthesis and analysis: The data will be synthesised narratively and meta-analysis will not be carried out. Finding will be reported in conjunction with the ‘Synthesis Without Meta-Analysis (SWiM)’ guideline where possible. ^32^ The summary of key study characteristics, PA evaluation methods will be tabulated. The outcome measures will be categorised and summarised as self-reported or clinician administered. The psychometric properties of each outcome measure with the statistical values will be summarised. We will summarise the practical considerations for each of the PA evaluation methods as per the clinical and research judgement of the investigator team and existing guidelines. ^11^^,^^33^ As the data are not related to treatment effectiveness, a summary of findings via GRADE methodology will not be included.

## Discussion

People with bronchiectasis often have reduced PA when compared to healthy age-matched peers ^7^^,^^12^ In order to assess the PA in adults with bronchiectasis, a list of suitable PA measurement tools is an ideal resource. This list can facilitate the PA assessment in variety of settings like physician’s office, pulmonary rehabilitation centre, hospital, home and occupational setting. This systematic review aims to list all the PA measures used in adults with bronchiectasis. Further, the review aims to list the measurement properties of each tool and quality of the studies that evaluated the measurement properties. Description on ease of use and time required to complete will be summarised, if available. These details will help clinicians to choose outcome measure that will be most feasible in terms of treatment, available resources, cost, time and the settings.

The PA assessment tools can be broadly classified as self-reported or clinician administered tools. Self-reported PA assessment tools are low cost, easy to administer and low burden method. ^33^ These measurements are often carried out at one time point and may be affected by recall and social desirability bias. ^33^ Although these self-reported tools are simple, easy to use and allow documentation of time spent on specific domain of PA, they may under or overestimate the PA. ^34^ Additionally, the self-reported PA assessment tools that are not primarily developed to assess the individuals with relatively limited amount of PA may not assess the domains of PA these individuals are involved in. Nevertheless, self-reported PA assessment tools can be of great utility in resource limited settings owing to low cost. ^33^ Alternatively, clinician administered outcome measure provide accurate, objective and are less susceptible to bias. However, they are relatively expensive and may involve data reduction and transformation process at the end. ^33^

By assessing the quality of studies on measurement properties, this review will help researchers choose the outcome measure for their evaluation of the effects of interventions on PA. This review will identify the suite of outcome measures of PA for people with bronchiectasis that can be used for research and clinical purpose.

### Study status

Piloting of the study selection process.

## CRediT author statement

Anup Bhat: Conceptualization, methodology, software, writing – Original draft.

Annemarie L Lee: Conceptualization, validation, Methodology, Writing – Review & Editing, Supervision.

Arun G Maiya: Conceptualization, Writing – Review & Editing, Supervision.

Vaishali K: Conceptualization, Methodology, validation, Writing – Review & Editing, Supervision.

## Data Availability

No underlying data are associated with this article. Figshare: PRISMA-P for Measurement properties of physical activity in adults with bronchiectasis: a systematic review protocol. DOI: https://doi.org/10.6084/m9.figshare.23599395.v1 ^35^ Data are available under the terms of the Creative Commons Attribution 4.0 International license (CC-BY 4.0).

## References

[1] King PT : The pathophysiology of bronchiectasis. *Int. J. Chron. Obstruct. Pulmon. Dis.* 2009;4:411–419. 20037680 10.2147/copd.s6133 PMC2793069

[2] Polverino E Dimakou K Hurst J : The overlap between bronchiectasis and chronic airway diseases: state of the art and future directions. *Eur. Respir. J.* 2018;52(3):1800328. 10.1183/13993003.00328-2018 30049739

[3] Ozalp O Inal-Ince D Calik E : Extrapulmonary features of bronchiectasis: muscle function, exercise capacity, fatigue, and health status. *Multidiscip. Respir. Med.* 2012;7(1):3. 10.1186/2049-6958-7-3 22958327 PMC3415114

[4] Gale NS Bolton CE Duckers JM : Systemic comorbidities in bronchiectasis. *Chron. Respir. Dis.* 2012;9(4):231–238. 10.1177/1479972312459973 23129800

[5] Bradley JM Wilson JJ Hayes K : Sedentary behaviour and physical activity in bronchiectasis: a cross-sectional study. *BMC Pulm. Med.* 2015;15:61. 10.1186/s12890-015-0046-7 25967368 PMC4456779

[6] Caspersen CJ Powell KE Christenson GM : Physical activity, exercise, and physical fitness: definitions and distinctions for health-related research. *Public Health Rep.* 1985;100(2):126–131. 3920711 PMC1424733

[7] José A Ramos TM Castro RAS de : Reduced Physical Activity With Bronchiectasis. *Respir. Care.* 2018;63(12):1498–1505. 10.4187/respcare.05771 30254043

[8] Lavery K Neill B Elborn JS : Self-management in bronchiectasis: the patients’ perspective. *Eur. Respir. J.* 2007;29(3):541–547. 10.1183/09031936.00057306 17079260

[9] Royle H Kelly C : ‘The likes of me running and walking? No chance’: Exploring the perceptions of adult patients with bronchiectasis towards exercise. *Chronic Illn.* 2022;19:157–171. 10.1177/17423953221108223 35695195 PMC9843538

[10] Dudgeon EK Crichton M Chalmers JD : “The missing ingredient”: the patient perspective of health related quality of life in bronchiectasis: a qualitative study. 10.1186/s12890-018-0631-7 PMC5964675 29788953

[11] Kelly CA Tsang A Lynes D : ‘It’s not one size fits all’: a qualitative study of patients’ and healthcare professionals’ views of self-management for bronchiectasis. *BMJ Open Respir. Res.* 2021;8(1):e000862. 10.1136/bmjresp-2020-000862 33664124 PMC7934710

[12] Cakmak A Inal-Ince D Sonbahar-Ulu H : Physical activity of patients with bronchiectasis compared with healthy counterparts: A cross-sectional study. *Heart Lung.* 2020;49(1):99–104. 10.1016/j.hrtlng.2019.09.004 31530430

[13] O’Neill B McDonough SM Wilson JJ : Comparing accelerometer, pedometer and a questionnaire for measuring physical activity in bronchiectasis: a validity and feasibility study? *Respir. Res.* 2017;18(1):16. 10.1186/s12931-016-0497-2 28088206 PMC5237513

[14] Alcaraz-Serrano V Gimeno-Santos E Scioscia G : Association between physical activity and risk of hospitalisation in bronchiectasis. *Eur. Respir. J.* 2020;55(6):1902138. 10.1183/13993003.02138-2019 32184321

[15] Alcaraz-Serrano V Arbillaga-Etxarri A Oscanoa P : Exacerbations and Changes in Physical Activity and Sedentary Behaviour in Patients with Bronchiectasis after 1 Year. *J. Clin. Med.* 2021;10(6):1190. 10.3390/jcm10061190 33809173 PMC7998500

[16] Sylvia LG Bernstein EE Hubbard JL : Practical guide to measuring physical activity. *J. Acad. Nutr. Diet.* 2014;114(2):199–208. 10.1016/j.jand.2013.09.018 24290836 PMC3915355

[17] Murphy J Uttamlal T Schmidtke KA : Tracking physical activity using smart phone apps: assessing the ability of a current app and systematically collecting patient recommendations for future development. *BMC Med. Inform. Decis. Mak.* 2020;20(1):17. 10.1186/s12911-020-1025-3 32013996 PMC6998214

[18] Troosters T Gosselink R Janssens W : Exercise training and pulmonary rehabilitation: new insights and remaining challenges. *Eur. Respir. Rev.* 2010;19(115):24–29. 10.1183/09059180.00007809 20956162 PMC9491643

[19] Pehlivan E Niksarlıoğlu EY Balcı A : The Effect of Pulmonary Rehabilitation on the Physical Activity Level and General Clinical Status of Patients with Bronchiectasis. *Turk. Thorac. J.* 2019;20(1):30–35. 10.5152/TurkThoracJ.2018.18093 30664424 PMC6340688

[20] José A Holland AE Selman JPR : Home-based pulmonary rehabilitation in people with bronchiectasis: a randomised controlled trial. *ERJ Open Res.* 2021;7(2):00021–02021. 10.1183/23120541.00021-2021 34084777 PMC8165366

[21] Lee AL Gordon CS Osadnik CR : Exercise training for bronchiectasis. *Cochrane Database Syst. Rev.* 2021;4(4):CD013110-CD. 10.1002/14651858.CD013110.pub2 33822364 PMC8093919

[22] Page MJ McKenzie JE Bossuyt PM : The PRISMA 2020 statement: an updated guideline for reporting systematic reviews. *BMJ.* 2021;372:n71. 10.1136/bmj.n71 33782057 PMC8005924

[23] Moher D Shamseer L Clarke M : Preferred Reporting Items for Systematic Review and Meta-Analysis Protocols (PRISMA-P) 2015 statement. *Syst. Rev.* 2015;4(1):1. 10.1186/2046-4053-4-1 25554246 PMC4320440

[24] Aliberti S Goeminne PC O’Donnell AE : Criteria and definitions for the radiological and clinical diagnosis of bronchiectasis in adults for use in clinical trials: international consensus recommendations. *Lancet Respir. Med.* 2022;10(3):298–306. 10.1016/S2213-2600(21)00277-0 34570994

[25] Mokkink LB Terwee CB Patrick DL : The COSMIN study reached international consensus on taxonomy, terminology, and definitions of measurement properties for health-related patient-reported outcomes. *J. Clin. Epidemiol.* 2010;63(7):737–745. 10.1016/j.jclinepi.2010.02.006 20494804

[26] Terwee CB Jansma EP Riphagen II : Development of a methodological PubMed search filter for finding studies on measurement properties of measurement instruments. *Qual. Life Res.* 2009;18(8):1115–1123. 10.1007/s11136-009-9528-5 19711195 PMC2744791

[27] Ouzzani M Hammady H Fedorowicz Z : Rayyan—a web and mobile app for systematic reviews. *Syst. Rev.* 2016;5(1):210. 10.1186/s13643-016-0384-4 27919275 PMC5139140

[28] Critical Appraisal Skills Programme: CASP cohort Checklist.Accessed: 02 May 2023. Reference Source

[29] *PEDro: Physiotherapy Evidence Database.* Australia: School of Public Health, University of Sydney Institute for Musculoskeletal Health;2019 [cited 02 May 2023]. Reference Source

[30] Mokkink LB Vet HCW de Prinsen CAC : COSMIN Risk of Bias checklist for systematic reviews of Patient-Reported Outcome Measures. *Qual. Life Res.* 2018;27:1171–1179. 10.1007/s11136-017-1765-4 29260445 PMC5891552

[31] Mokkink LB Boers M Vleuten CPM van der : COSMIN Risk of Bias tool to assess the quality of studies on reliability or measurement error of outcome measurement instruments: a Delphi study. *BMC Med. Res. Methodol.* 2020;20(1):293. 10.1186/s12874-020-01179-5 33267819 PMC7712525

[32] Campbell M McKenzie JE Sowden A : Synthesis without meta-analysis (SWiM) in systematic reviews: reporting guideline. *BMJ.* 2020;368:l6890. 10.1136/bmj.l6890 31948937 PMC7190266

[33] Strath SJ Kaminsky LA Ainsworth BE : American Heart Association Physical Activity Committee of the Council on Lifestyle and Cardiometabolic Health and Cardiovascular, Exercise, Cardiac Rehabilitation and Prevention Committee of the Council on Clinical Cardiology, and Council. Guide to the assessment of physical activity: Clinical and research applications: a scientific statement from the American Heart Association. *Circulation.* 2013;128(20):2259–2279. 10.1161/01.cir.0000435708.67487.da 24126387

[34] Ainsworth B Cahalin L Buman M : The current state of physical activity assessment tools. *Prog. Cardiovasc. Dis.* 2015;57(4):387–395. 10.1016/j.pcad.2014.10.005 25446555

[35] Bhat A Lee A Maiya GA : PRISMA-P for Measurement properties of physical activity in adults with bronchiectasis: a systematic review protocol. figshare. *Figure.* 2023. (35). 10.6084/m9.figshare.23599395.v1 PMC10439356 37600906

