# Supplementary material for: Measurement properties of physical activity in adults with bronchiectasis: A systematic review protocol
Source: F1000Res. 2023 Jul 10;12:801. [Version 1] doi: 10.12688/f1000research.138593.1 (PMC10439356; doi:10.12688/f1000research.138593.1)
Supplement: Peer review history

# Peer review: Reviewer response for version 1

**DOI:** 10.5256/f1000research.151802.r186195

**Published:** 2023-08-18

**Authors:** Vishnu Vardhan G. D.

**Affiliations:** 1 Cardiorespiratory Physiotherapy, Ravi Nair Physiotherapy College, Datta Meghe Institute of Medical Sciences, Wardha, Maharashtra, India

The paper is about bronchiectasis, a chronic airway disease that causes chronic cough with expectoration and recurrent exacerbations. People with bronchiectasis experience major clinical features such as muscular weakness, loss of muscular endurance, dyspnea, fatigue, and poor quality of life. Due to these symptoms, they often avoid physical activities, which further decondition them. The paper aims to identify and evaluate the literature on psychometric properties of instruments measuring and analyzing physical activity in adults with bronchiectasis.

1. The title needs to be modified.
2. Age criteria of patients of bronchiectasis with less physical activity to be mentioned in Introduction.
3. Qualitative studies may be included in inclusion criteria.
4. Mention the time duration needed for the review in methods.
5. Mention the year range for selecting the 5 articles in methods.

Is the study design appropriate for the research question?

Yes

Is the rationale for, and objectives of, the study clearly described?

Yes

Are sufficient details of the methods provided to allow replication by others?

Yes

Are the datasets clearly presented in a useable and accessible format?

Yes

Reviewer Expertise:

NA

I confirm that I have read this submission and believe that I have an appropriate level of expertise to confirm that it is of an acceptable scientific standard.

# Peer review: Reviewer response for version 1

**DOI:** 10.5256/f1000research.151802.r186191

**Published:** 2023-07-18

**Authors:** Jaya Shanker Tedla

**Affiliations:** 1 Department of Medical Rehabilitation Sciences, College of Applied Medical Sciences, King Khalid University, Abha, Aseer Province, Saudi Arabia

The method paper reads well. Currently, a plethora of physical activity assessment tools are available. This protocol has the potential to address and summarize the available physical activity assessment tools. The following concerns need to be addressed:

1. The title needs to be modified to “Measurement properties of physical activity assessment tool in adults with bronchiectasis: A systematic review protocol.”
2. Authors can introduce the prevalence and economic impact of bronchiectasis in the introduction.
3. In the eligibility criteria, what about case reports and case series?
4. Domain-specific physical activity can be discussed in the introduction.
5. In the exclusion criteria, qualitative studies are excluded. Qualitative analysis could be one of the steps involved in developing some outcome measures. Would you like to include such studies?
6. What is the procedure if full texts are not available?
7. Quality assessment tool for qualitative studies.
8. The discussion section is important for a full review. The inclusion of this in the method paper is a plus point.
9. Authors can discuss remote monitoring of PA using available tools.

Is the study design appropriate for the research question?

Yes

Is the rationale for, and objectives of, the study clearly described?

Yes

Are sufficient details of the methods provided to allow replication by others?

Yes

Are the datasets clearly presented in a useable and accessible format?

Yes

Reviewer Expertise:

My expertise is in physical therapy and rehabilitation

I confirm that I have read this submission and believe that I have an appropriate level of expertise to confirm that it is of an acceptable scientific standard.
